# Properties Enhancement Nano Coconut Shell Filled in Packaging Plastic Waste Bionanocomposite

**DOI:** 10.3390/polym14040772

**Published:** 2022-02-16

**Authors:** Ismail Ismail, Quratul Aini, Zulkarnain Jalil, Niyi Gideon Olaiya, Mursal Mursal, C.K. Abdullah, Abdul Khalil H.P.S.

**Affiliations:** 1 Physics Department, Mathematics and Natural Sciences Faculty, Universitas Syiah Kuala, Banda Aceh 23111, Indonesia; quratulainifisika@gmail.com (Q.A.); zjalil@unsyiah.ac.id (Z.J.); mursal@unsyiah.ac.id (M.M.); 2 School of Industrial Technology, Universiti Sains Malaysia, Penang 11800, Malaysia; ngolaiya@futa.edu.ng (N.G.O.); ck_abdullah@usm.my (C.K.A.)

**Keywords:** biocomposite, mechanical properties, physical properties, polypropylene, thermal properties

## Abstract

Plastic waste recycling has been proposed as a long-term solution to eliminate land and marine deposit. This study proposed a new approach to fabricate biocomposites of nano-sized fillers and low matrix compositions with a great performance by using plastic packaging waste different from the conventional biocomposite. Coconut shell, an agricultural waste, was bonden with waste plastic to form a biocomposite with a coupling agent. The optimum percentage composition and the effect of coconut shell ball milling time on the properties of the biocomposite were studied with density, thickness swelling, porosity flexural strength, flexural modulus, compressive strength, thermogravimetric analysis, differential scanning calorimetry, scanning electron microscope (SEM), and atomic force microscopy (AFM). The results showed that the optimum performance of biocomposite was obtained at 30/70 (wt.%) plastic waste to coconut shell ratio, where 70 wt.% was the highest coconut shell composition that can be achieved. Furthermore, for 30 wt.% of polypropylene (low matrix), the performance of biocomposite improved significantly with milling time due to enhanced interaction between filler and matrix. As the milling time was increased from 0 to 40 h, the density increased from 0.9 to 1.02 g/cm^3^; thickness swelling decreased from 3.4 to 1.8%; porosity decreased from 7.0 to 3.0%; flexural strength increased from 8.19 to 12.26 MPa; flexural modulus increased from 1.67 to 2.87 GPa, and compressive strength increased from 16.00 to 27.20 MPa. The degradation temperature of biocomposite also increased as the milling duration increased from 0 to 40 h. The melting temperature increased significantly from 160 to 170 °C as the milling duration increased from 0 to 40 h. The depolymerisation occurred at 350 °C, which also increased with milling duration. This study revealed that the performance of biocomposite improved significantly with a lower percentage matrix and fillernanoparticle rather than increasing the percentage of the matrix. The nanocomposite can be used as a panelboard in industrial applications.

## 1. Introduction

The use of composite materials has been increased significantly. Many composites applications include furniture, household appliance, electronic device, automotive, aircraft, etc. [1]. Consequently, composite materials have gained interest among researchers and industries [2,3]. As it is known, composite materials are new materials resulting from combining two or more compatible materials. One material is called the dispersed phase (filler), and the other is the matrix phase (adhesive). Conventionally, composite fillers are synthetic materials, such as fibreglass, carbon, silica carbide, aramid [1]. Synthetic composite fillers have a high performance, such as strong but light-weight. However, they are not good for the environment, are not eco-friendly, cause microplastic pollution, and are not sustainable. Because of the environmental concern, scientists are developing composites by using sustainable fillers that are derived from natural fibres (flax, sisal, kenaf, bamboo), agro or forestry residues (rice straw, coconut shell, coconut coir, wheat, corn), recycled fillers (carpet, cardboard, carbon), and industrial co-products (baggase, grape pomade, lignin, etc.) [4,5]. Those sustainable fillers can be used to reinforce polymer matrices (such as epoxy, polypropylene, polyethene) to form composites, called biocomposites [6,7]. Biocomposite research is in great demand today to address environmental issues [8,9]. Recently, biodegradable spoons were successfully fabricated from the mixture of grape seed, wheat, millet, and xanthan [10]. To produce a high-quality biocomposite from natural fibres is a big challenge because of the compatibility between biofibres and polymers [11]. Biofibre (filler) is a hydrophilic material, while polymer (matrix) is hydrophobic, which causes poor bonding between filler and matrix. An alternative solution was to use a coupling agent (chemical treatment) to improve the compatibility between the filler and matrix, which may improve the performance of biocomposites [12]. Nonetheless, it is very challenging to have good properties of biocomposite for the high load of fillers, less composition of the matrix.

One of the abundant agricultural waste materials (biofibres) in tropical countries is coconut shells. Science agriculture reported that the production of coconut worldwide was about 61 million tons in 2019 [13]. Coconut shell contains 34% cellulose, 21% hemicellulose, and 27% lignin [14]. Previous studies showed that the coconut shell was potentially a filler to form a composite [14,15,16]. Singh formed a coconut shell composite using epoxy resin. The size of particles was 212–850 µm. The compositions of coconut shell particles were 20, 30, 40 wt.%. The mechanical properties of the composite decreased as for the composition 40 wt.% of fillers [15]. Bhaskar studied composite made of coconut shell particles (200–800 µm) and epoxy resin. The compositions of coconut shells were 20, 25, 30, and 35 wt.%. They reported that the ultimate strength and modulus of elasticity of composites decreased as the compositions of coconut shells were increased [16]. There were a number of other studies on coconut shell composites [17,18,19,20,21,22]. However, the compositions of fillers for most biocomposites were below 50% and micrometre of filler sizes. On the other hand, it is expected to have a good performance of biocomposite for high loading of fillers, less matrix to use more sustainable resources (biofibres). 

Plastic pollution is one of the major environmental issues. The amount of plastics used in the world is increasing every year. In 1950, the total plastic production in the world was about 2.3 million tonnes. It increased to about 448 million tons in 2015. Plastic production worldwide will be around 900 million tons in 2050 [23]. According to MacArthur, about 8 million tonnes of plastic wastes enter the ocean every year [24]. This number will increase following the increase in plastic production in the world. There are several types of plastic waste. One of them is polypropylene (code 05). Polypropylene is a thermoplastic polymer widely used in various applications, such as product packaging [25].

Therefore, waste polypropylene around us should be utilised to overcome the environmental issue. The polypropylene from plastic waste can be recycled as a matrix to mix with sustainable fillers in forming the biocomposite. However, few studies on biocomposites use waste plastic (polypropylene) as an adhesive. Chun et al. prepared a composite from coconut shell particles and recycled polypropylene. The size of the filler was 70 mesh, and the composition of the coconut shell was 0–40% [26]. It was found that the composition of fillers significantly affected the mechanical and thermal properties of the composite. However, the polypropylene composition from those studies was large, i.e., 60–100%. The physical properties of the composite were not reported [26]. Agunsoye also studied the recycled polypropylene reinforced with the coconut shell using coconut shell particles (100 and 200 mesh). However, the filler composition (coconut shell particles) was only 5–25%, i.e., 75–95% polypropylene. The impact energy decreased as the composition of coconut shell particles was above 15%. However, they observed that the impact energy for small particles (200 mesh) was higher than 100 mesh for certain compositions [19]. Thus, it is interesting to study the effect of particle (filler) sizes on the properties of biocomposites.

Several previous studies have shown that nanometer-sized fillers can improve the performance of composites [20,27,28,29,30]. Ball milling can be applied to produce nanoparticles or nanopowders [31]. Duration of milling or milling time is one of the important parameters in producing nanoparticles [32]. The milling duration significantly affected the composites’ properties [32,33,34]. A recent study showed that nanoparticles of coconut shell mixed with epoxy resin improved the biocomposite performance [32]. There is no study about nanocomposite or nano board made of coconut shell and the waste plastic packaging as matrix found in the literature. Meanwhile, it is very important to utilise plastic waste to overcome the environmental issue. Other than that, nano board or nanocomposite has a very high prospect of developing biocomposites.

The present work is to prepare a biocomposite of coconut shell particles with a new approach. The coconut shell particles were added into the polypropylene matrix from the packing-plastic waste to form the biocomposite. Benzoyl peroxide, xylene, methanol, and anhydride maleic were used as the coupling agent to improve the compatibility between coconut shell particles and polypropylene. The size of coconut shell particles was reduced to nano-size to improve the performance of the biocomposite. The physical, mechanical, thermal, and morphological properties of biocomposite were examined.

## 2. Materials and Methods

### 2.1. Materials

The waste plastic bottle packaging for mineral water (polypropylene, code 5, PP) was collected from the recycled plastic place in Banda Aceh, Indonesia. The waste plastic was cleaned and cut by about 1 cm × 1 cm. PT Indratma Sahitaguna, Indonesia, supplied the coconut shell powder having 200 mesh sizes. Benzoyl peroxide (luperox A70S, 632651), xylene (reagent grade, 214736), methanol (gradient grade, 34885), maleic anhydride (99%, M188) were purchased from Sigma Aldrich, Indonesia.

### 2.2. Sample Preparation

**Figure 1:**
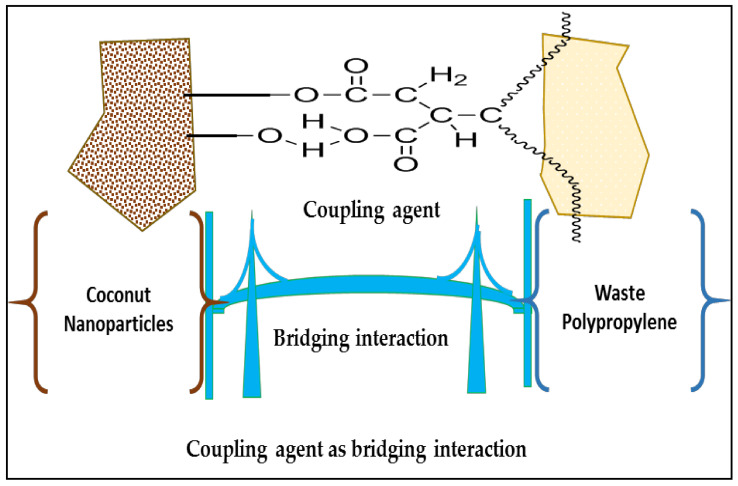
Schematic of possible coupling agent bridging interaction in the biocomposite.

The coconut shell particles with particle size 200 mesh, waste plastic packaging (recycled polypropylene dissolved in xylene as solvent), and coupling agent (benzoyl peroxide (initiator), methanol (reaction solvent), anhydride maleic (modifier)) were mixed using a rheomixer (manufactured by Polytechnic, Medan, Indonesia) at 60 °C for 60 min [35,36]. The chamber volume of the mixer was 625 cm^3^, equipped with electrical heating with atemperature up to 350 °C. Figure 1 shows the possible coupling agent reaction in the biocomposite. 

The sample mixture was transferred into the extrusion (manufactured by Polytechnic, Medan, Indonesia) heated at 180 °C with 30 rpm of the rotor speed. After that, the mixture was poured into the compression moulding machine and pressed with 3 tons of load for 1 h at 175 °C to obtain a biocomposite sample. The mould was made of steel with 200 mm × 150 mm. The moulding machine (manufactured by Polytechnic, Medan, Indonesia) was equipped with an electric heater a hydraulic press with a load of up to 4 tons. The temperature can be controlled automatically, up to 350 °C.

The composition of biocomposite samples for 200 mesh of coconut shell particles (CSP) is listed in Table 1. The composition of polypropylene (PP) from packaging plastic waste was varied from 30 to 100 wt.%. The size of the biocomposite sample fabricated was 200 mm × 150 mm × 10 mm. The samples were stored in a dried place to prevent moisture. 

To study the effect of particle size and milling time on the properties of the biocomposite, the coconut shell powder (200 mesh) was milled at room temperature by a ball mill to produce nanoparticles. The ball mill used in this study was a planetary type, Planetary Mono Mill Pulverisette 6 manufactured by Fritsch Germany, consisting of one working station. The grinding bowl size was 250 mL with a grinding ball diameter of 15 mm. The ratio of ball to powder was 10:1 (wt.). The milling was conducted at room temperature with a rotational speed of 350 rpm (constant speed) and a dry process. The milling duration was varied from 0, 10, 20, 30 to 40 h. The resulting crystallite nanoparticle of the coconut shell was confirmed with X-ray diffraction and Transmission Electron Microscopy (TEM) [32]. The coconut shell nanoparticle was used in biocomposite with propylene waste at an optimum percentage obtained from the composition variation and studied properties.

### 2.3. Characterisation of the Biocomposite

The testing of the physical properties of samples was conducted according to the Indonesian National Standard for particleboard [37]. The density of the composite sample (*ρ*) was obtained using the Equation (1).

(1) $$\rho =\frac{m}{V}$$

where *V* is the volume of the sample; *m* is the mass of the sample. The porosity of the sample (*PR*) was determined using Equation (2).

(2) $$PR=\frac{m_{wet}-m_{dry}}{V_{bulk}}\times \frac{1}{\rho_{water}}\times 100\%$$

where *V_bulk_* is sample volume; *ρ_water_* is the density of water; *m_dry_* and *m_wet_* are the mass of the sample before and after the sample was immersed in the water for 24 h. The sample’s thickness swelling (TSW) percentage was calculated using Equation (3).

(3) $$TSW=\frac{T_{wet}-T_{dry}}{T_{dry}}\times 100\%$$

where *T_dry_* is the sample thickness before immersing in the water; *T_wet_* is the thickness of the sample after the sample was immersed in the water for 24 h. 

The mechanical properties of biocomposite were determined according to the Indonesian National Standard for particleboard [37]. The measurement was conducted using the universal testing machine manufactured by Hung Ta Company (Taiwan). The flexural strength (*FS*) was obtained using Equation (4).

(4) $$FS=\frac{3\times B\times S}{2\times L\times T^{2}}$$

where *B* is the maximum load; *S* is the span; *T* is the sample thickness; *L* is the sample width. The flexural modulus (*FM*) was calculated using Equation (5).

(5) $$FM=(\frac{\Delta B}{\Delta D})\frac{S^{3}}{4\times L\times T^{3}}$$

where *S*, *L*, and *T* are the same as in Equation (4); (Δ*B/*Δ*D*) is the slope of the force to deformation. The compressive strength (*CS*) was determined using Equation (6).

(6) $$CS=\frac{F_{\max}}{A}$$

where *F*_max_ is the load at the point of failure, and *A* is the cross-sectional area of the sample. 

The thermogravimetric analysis (TGA) and differential scanning calorimetry (DSC) was utilised to determine the thermal properties of the biocomposite samples. The TGA equipment was produced by Shimadzu, type DTG–60 (Shimadzu, Kyoto, Japan). The DSC equipment was manufactured by Shimadzu, type DSC–60 (Shimadzu, Kyoto, Japan). The heating rate of the sample for both DSC and TGA measurements was 10 °C per minute.

Atomic force microscopy (AFM) (Nanosurf, Liestal, Switzerland) and a scanning electron microscope (SEM) (Thermo Fisher Scientific, Waltham, MA, USA) have been used to evaluate the morphological properties of composite samples. The sample size was 1 cm × 1 cm with 1 cm thickness. The sample was put on the carbon conductive double tape where the tape was stuck on the stub. Then, the samples were gold coated, and the stub with the sample on it was inserted into the chamber. The SEM chamber was operated at the high vacuum mode.

### 2.4. Statistical Analysis

One-way analysis of variance (ANOVA) with Duncan’s multiple range tests was used to test the homogeneity of variance and determine the effect of PP composition and duration of milling on the physical (Table A1 and Table A3) and mechanical (Table A2 and Table A4) properties of biocomposite. The statistical significance used in the analysis was 0.05 (5%). The calculation was conducted by using the SPSS software version 16.0 (IBM, Chicago, IL, USA).

## 3. Results and Discussion

### 3.1. Properties of Biocomposite with Varying Composition

The biocomposite from coconut shell particles using the plastic waste (recycled polypropylene) as the matrix has been successfully prepared. The possible schematic bonding between the biocomposite materials is shown in Figure 1. The coupling agent formed a bridging effect between the coconut shell and the propylene. This enhances the binding efficiency of propylene in the biocomposite. Previous studies reported the bridging effect of the coupling agent used in this study [36,38,39,40].

#### 3.1.1. Physical Properties

**Figure 2:**
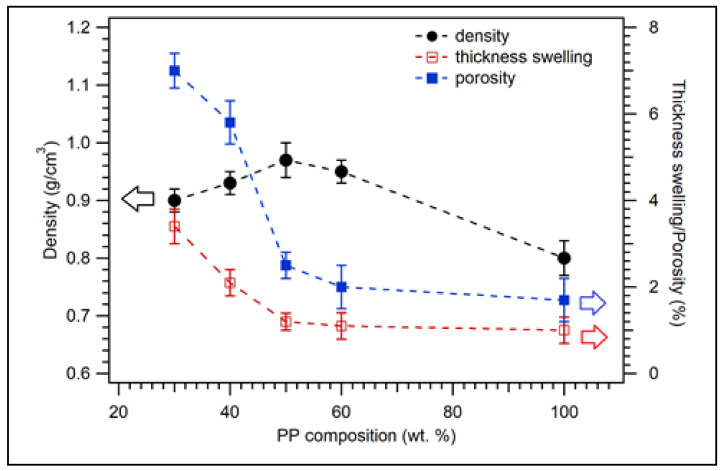
Physical properties of coconut shell biocomposite for various PP compositions with 200 mesh of coconut shell particles.

The density, thickness, swelling, and porosity of biocomposite with coconut shell for various compositions of polypropylene (PP) are shown in Figure 2. For the composition of PP 30 wt.%, composite density was 0.90 g/cm^3^. The density increased to 0.97 g/cm^3^ for PP 50 wt.%. The increasing density of composite from 30 to 50 wt.% of PP (see filled circles in Figure 2) could be related to the well-blended between PP and coconut shell particle at the ratio composition of 50/50 wt.%. However, the density decreased to 0.95 and 0.85 g/cm^3^ for 60 and 100 wt.%, respectively. The decreasing density of composite for the composition of PP above 50 wt.% is because the density of PP is low; i.e., the density of virgin PP is 0.855 g/cm^3^. The highest composite density from the present study was 0.97 g/cm^3^, which occurred at 50 wt.% coconut shell and 50 wt.% PP. The density of composite for 75 wt.% HDPE (25 wt.% coconut shell particle) is about 0.91 g/cm^3^ [41], almost the same density as this study. 

The thickness swelling of biocomposite for various PP compositions is displayed in Figure 2 (unfilled squares). For 30 wt.% of PP, the thickness swelling of the composite was 3.4%. The thickness swelling of the sample decreased to 1.1% and 1.0% for the composition of PP 60 and 100 wt.%, respectively. The thickness swelling of the composite decreased significantly as the composition of PP was increased because PP is hydrophobic (repelling water). Figure 2 (filled squares) shows the porosity of biocomposite samples for various PP compositions with 200 mesh particle sizes. The porosity of the sample for 30 wt.% of PP is 7%. For 40 wt.% of PP, the porosity decreased to 5.8%. The porosity of the composite continued decreasing as the composition of PP was increased. The porosity was 1.7% for 100 wt.% of PP. The trend of porosity is the same as that of thickness swelling. In general, the physical properties (thickness swelling and porosity) of coconut shell biocomposite improved as the composition of PP was increased. Nonetheless, it is expected that the composition of PP should be minimised (low matrix) for biocomposites.

The results of statistical analysis of physical properties of biocomposite with various PP compositions are listed in Table A1. The statistically significant values of density, thickness swelling, and porosity measurements are 0.740, 0.434, and 0.483, respectively. These numbers are larger than 0.05, indicating that the variances of density, thickness swelling, and porosity data are homogenous. The F values from the calculation for density, thickness swelling, and porosity are 21.800, 54.138, and 155.353, respectively. The value of F theoretical (5%) for df 4/20 is 2.87. The value of F calculation is larger than the values of F theoretical, which indicates the physical properties of biocomposite are significant differences for different PP compositions. This finding confirms a significant effect of PP composition on the physical properties of biocomposite.

#### 3.1.2. Mechanical Properties

**Figure 3:**
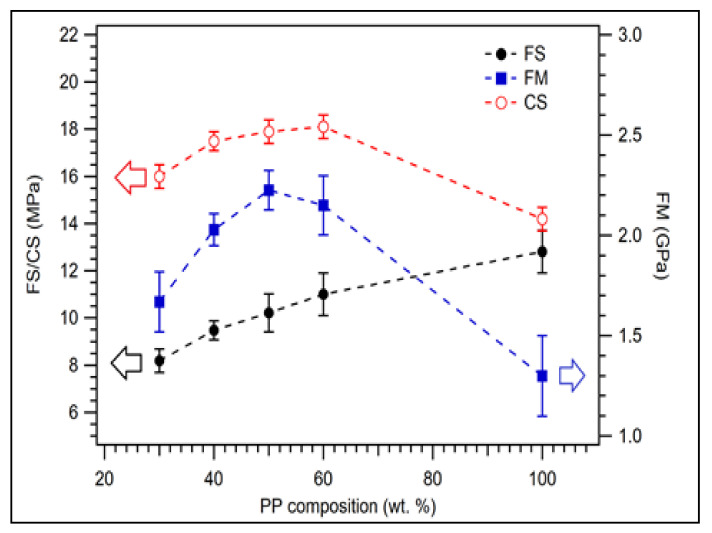
Flexural properties of coconut shell biocomposite for various PP compositions with 200 mesh of coconut shell particles.

The flexural strength, flexural modulus, and compressive strength of coconut shell biocomposite weremeasured. Figure 3 displays the flexural strength, flexural modulus, and compressive strength of biocomposite samples for 200 mesh particle size with various PP compositions. As the composition of PP was increased, it was found that the flexural strength (FS) of the composite increased from 8.19 MPa for 30 wt.% of PPto 11.00 MPa for 60 wt.% of PP, see filled circles in Figure 3. For 100 wt.% of PP, the flexural strength increased to 12.80 MPa. The FS for 100 wt.% PP is significantly lower than the value of FS for virgin PP is 32 MPa [42].

The flexural modulus of composite was 1.67 GPa for 30 wt.% of PP. Its value increased to 2.23 GPa for 50 wt.% of PP. However, the flexural modulus slightly decreased to 2.15 GPa for 60 wt.% compositions of PP. The flexural modulus was 1.30 GPa for 100 wt.% PP. The highest flexural modulus of biocomposite was found at 50 wt.% PP, see filled square in Figure 3. For the composition of PP greater than 50 wt.%, the flexural modulus decreases because the flexural modulus of virgin PP is about 1.45 GPa [42]. The measured compressive strength is displayed in unfilled circles in Figure 3. Its value was 16.00 MPa for 30 wt.% of PP and increased to 18.10 MPa for 60 wt.% of PP. The compressive strength decreased to 14.20 MPa for 100 wt.% of PP. This value is lower than the value of virgin PP reported previously, which is 34.4 MPa [43]. The discrepancy between the results of this study and previous studies could be due to the purity of the matrix.

In general, this study revealed that the composition of PP significantly influenced the mechanical properties of the biocomposite. This behaviour was observed in the previous study where composite tensile strength and elongation break increased as the matrix (PP) composition increased [26]. The flexural strength, flexural modulus, and compressive strength of rice straw composite also increased as the composition of PP was increased [44]. By considering the best flexural modulus value, the good mechanical properties of coconut shell PP biocomposite were obtained at the 50 wt.% PP composition.

Table A2 displays the statistical analysis of mechanical properties of biocomposite with various PP compositions. The statistically significant values of flexural strength, flexural modulus, and compressive strength data are 0.540, 0.264, and 0.017, respectively, larger than 0.05. This indicates that the variances of flexural strength, flexural modulus, and compressive strength data are homogenous. The F values from the calculation for flexural strength, flexural modulus, and compressive strength are 27.407, 36.774, and 56.935, respectively, larger than the F theoretical value (2.87), which indicate the mechanical properties of biocomposite are significant differences for different PP compositions. This information confirms that PP compositions significantly influence the mechanical properties of biocomposites.

#### 3.1.3. Thermal Properties

**Figure 4:**
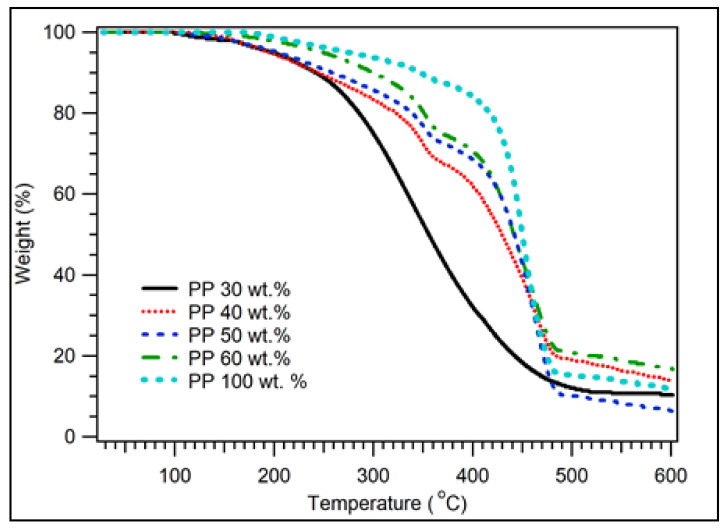
TGA of coconut shell biocomposite for various PP compositions with 200 mesh of coconut shell particles.

The thermal properties of the coconut shell biocomposites have been evaluated. Figure 4 displays the TGA of biocomposite samples with 200 particle size mesh for various PP compositions. The summary of decomposition temperature of biocomposite for various PP compositions is listed in Table 2. For 30 wt.% of PP, the decomposition temperature was 199 °C for 95% of the weight. At 242 °C, the remaining sample was 90%. The weight of the sample was still 80% at 286 °C. Above that temperature, decomposition significantly occurred. At the temperature of 356 °C, only 50% of thesample was left. The weight of the sample was 20% at 443 °C. The result obtained is almost identical to the previous study, where coconut shell powder’s degradation temperature is 250–450 °C [45]. As the composition of PP was increased, the decomposition of temperature increased, as shown in Figure 4 and Table 2. 

**Figure 5:**
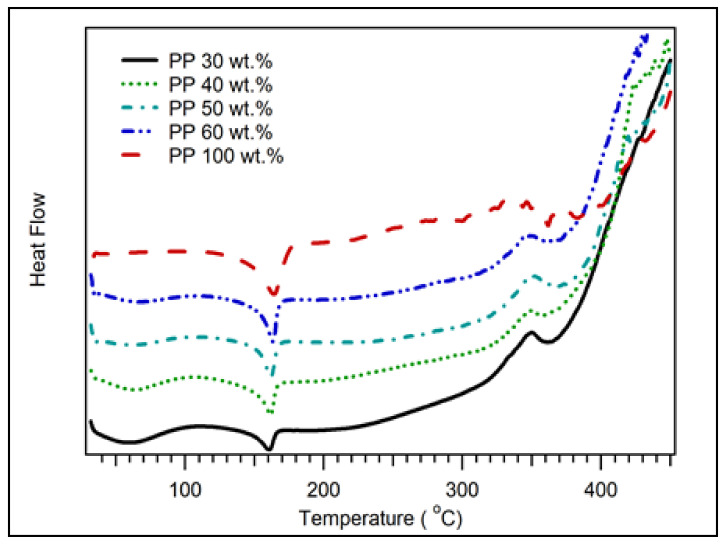
DSC of coconut shell biocomposite for various PP compositions with 200 mesh of coconut shell particles.

The DSC curves for the coconut shell biocomposites for various PP compositions are displayed in Figure 5. There was a broad peak observed at 60 °C. This peak was associated with coconut shell biocomposite’s glass transition temperature (Tg). As the composition of PP increased, the peak widened and disappeared at 100 wt.% of PP. The disappearance of the Tg peak at the 100% PP composition is due to the Tg of PP at a low temperature of about −10 °C. The observed Tg for this study was about the same as Chun et al. for coconut shell particle polylactic acid composite 63 °C [26]. The melting temperature (Tm) was observed around 162 °C. As the composition of PP increased, the Tm of biocomposite improved. Another peak was observed at 350 °C, which was related to depolymerisation. As shown in the TGA results, decomposition occurs drastically at 350 °C. The summary of DSC data of coconut shell biocomposite for various PP compositions is listed in Table 3. As the composition of PP increased, the Tg and Td of biocomposite decreased. However, its Tm increased from 160 to 164 °C.

#### 3.1.4. Morphological Properties

The morphology of biocomposite samples for 200 mesh particle size with the various compositions of PP was examined by SEM and AFM. The results are displayed in Table 4. The surface was rather rough for 30 wt.% compositions of PP (70 wt.% of coconut shell particles). There were some porosities and agglomerations observed. As the PP composition increased 50 wt.%, the coconut shell particles were mixed well with PP, reducing the porosities. The surface corrugation of biocomposite reduced as the composition of PP was increased, as shown by the AFM image in Table 4. The biocomposite becomes denser, so the density becomes greater. The bond between the filler and the adhesive becomes better, increasing the mechanical properties for 100 wt.% PP composition, the surface is homogenous and smooth.

### 3.2. Properties of Biocomposite with Varying Particle Size

The crystallite size and TEM images of the coconut shell particles for each milling time are shown in Table 5. The TEM images confirmed the production of nanoparticles from the coconut shell milling time. The particle size of the coconut shell reduces with milling time until 40 h. Further milling time has no significant effect on its particle size. The particle size of the coconut shell was found to be 48 nm for 10 h of milling time, which decreases with increasing milling duration, as displayed in filled circles in Table 5. The particle size decreases to 30 nm for a 40 h milling duration. The size of the coconut shell remains constant after 40 h which shows the optimum milling time. The trend of the result was similar to that found in the literature.

#### 3.2.1. Physical Properties

**Figure 6:**
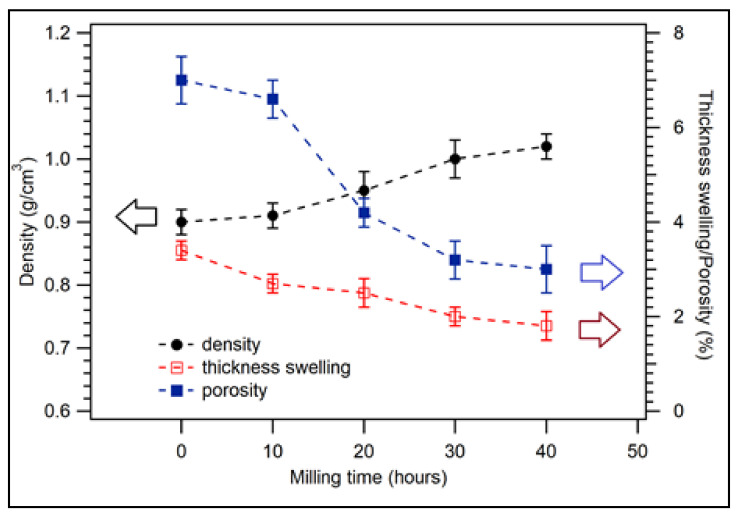
Physical properties of coconut shell nano-biocomposite for various milling times with 30 wt.% of PP composition.

For the composition of samples with 30 wt.% of recycled polypropylene and 70 wt.% of coconut shell particles, the milling duration was varied from 0, 10, 20, 30, to 40 h. The physical properties of nano-biocomposite are shown in Figure 6 for the 30 wt.% compositions of PP (70 wt.% of coconut shell particle). The density of biocomposite was found to be 0.91 g/cm^3^ for 10 h of milling time. Its density increased with increasing milling duration, as displayed in the filled circles in Figure 6. The density was 1.02 g/cm^3^ for a 40 h milling duration. The density of biocomposite found in this study is lower than that of coconut shell nanoparticles with epoxy resin (1.03–1.19 g/cm^3^). However, its trend is the same [32]. Figure 6 (unfilled squares) displays the thickness swelling of biocomposite for several milling durations. For 0 h duration of milling, the thickness swelling of biocomposite was 3.4%. As the milling time was increased, the thickness swelling decreased. For 40 h duration of milling, the thickness swelling reduced to 1.8%. Filled squares depict the porosity of biocomposites in Figure 6. For 10 h of milling time, the porosity of the composite sample was 6.6%. The porosity of the sample was reduced to 3.0% for 40 h. The porosity of the composite decreased with the increase of milling times which is the same trend for thickness swelling. The previous work also observed this behavior [32].

In general, the physical properties of the biocomposite improve significantly as the milling times increase or reduce the particle sizes. This property occurs due to the nano-sized of coconut shell [32]. Nano-sized filler can blend well with the matrix during the formation of composite, which causes the composite to be dense. As a result, the porosity of the composite decreases, which improves the density of the biocomposite. This study indicates that it is unnecessary to increase an adhesive or matrix composition to obtain good physical properties of biocomposite. Still, it can be achieved by reducing the particle size of filler from micro to nanometer. 

The statistical analysis of the physical properties of biocomposite with various durations of milling is listed in Table A3. The significant statistical values of density, thickness swelling, and porosity data are 0.170, 0.274, and 0.361, respectively, are larger than 0.05. This shows that the variances of density, thickness swelling, and porosity data for various milling times are homogenous. The calculated F values for density, thickness swelling, and porosity are 21.938, 28.571, and 117.353, respectively. The value of F theoretical (5%) for df 4/20 is 2.87. The calculated F values are larger than the values of F theoretical, which means the physical properties of biocomposite are significant differences for different duration of millings. This confirms that the milling duration affects the physical properties of biocomposite significantly.

#### 3.2.2. Mechanical Properties

**Figure 7:**
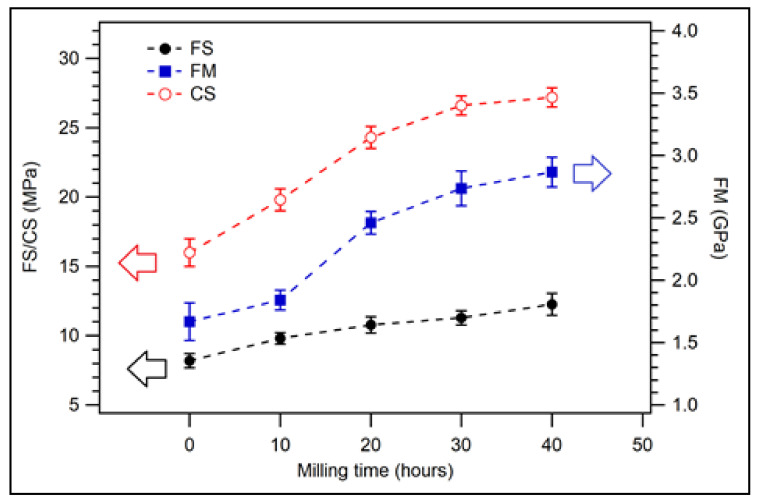
Mechanical properties of coconut shell nano-biocomposite for various milling times with 30 wt.% of PP composition.

Figure 7 displays the flexural strength, flexural modulus, and compressive strength of the biocomposite for various milling times with 30 wt.% of PP composition. For the 10 h duration of milling, the flexural strength was 9.81 MPa which was larger than the value for 0 h of milling (8.19 MPa). The flexural strength of biocomposite increased significantly with the increasing milling times, as depicted by the filled circles in Figure 7. For 40 h milling duration, the flexural strength of the composite was found to be 12.26 MPa which was larger than the value for 60 wt.% of PP. This finding shows that the flexural strength of coconut shell bio-nanocomposite can be improved significantly by increasing the duration of milling time without increasing the composition of PP.

Figure 7 shows the flexural modulus of coconut shell bio-nanocomposite samples for 30 wt.% of PP with various milling duration. For 10 h of milling, the flexural modulus of coconut shell bio-nanocomposite was 1.842 GPa. The flexural modulus value increases as the milling duration increases, as displayed in filled squares in Figure 7. For the duration of milling 40 h, the flexural modulus of coconut shell bio-nanocomposite was 2.767 GPa. Its compressive strength was also affected significantly by the duration of milling (see unfilled circles in Figure 7). The compressive strength increased from 16.0 MPa for 0 h of milling to 27.2 MPa for 40 h of milling.

Similar to the physical properties, the mechanical properties of biocomposite improve as the duration of milling increases. This implies that particle sizes (nanoparticles) play an important role in the properties of a composite [32]. As the coconut shell particles become smaller, the contact surface areas between the fillers and matrix (PP) increase. Consequently, the bonding between matrix and fillers increases [30]. Moreover, nanoparticles are well blended with the matrix. As a result, the physical and mechanical properties of the composite improve significantly. This behaviour is similar to that observed in the previous study (coconut shell–epoxy resin composite) [32].

This study’s highest flexural strength and modulus values are obtained at the 40 h milling time with 30 wt.% of PP 12.260 MPa and 2.767 GPa, respectively. The values are met the Indonesian National Standard for particleboard. The results obtained from this study are almost the same as the results from the previous study, a rice straw composite using PP as the matrix [14,44].

Table A4 shows the statistical analysis of mechanical properties of biocomposite with various milling times. The significant statistical values of flexural strength, flexural modulus, and compressive strength data are 0.305, 0.653, and 0.715, respectively, larger than 0.05. This means that the variances of flexural strength, flexural modulus, and compressive strength data for various milling durations are homogenous. The calculated F values for flexural strength, flexural modulus, and compressive strength with various milling times are 39.028, 102.017, and 228.771, respectively, larger than the F theoretical value (2.87). This finding indicates the mechanical properties of biocomposite are significant differences for different durations of milling (0, 10, 20, 30, and 40 h). The statistical results confirm that the mechanical properties of biocomposite are significantly influenced by the duration of milling or particle sizes.

#### 3.2.3. Thermal Properties

**Figure 8:**
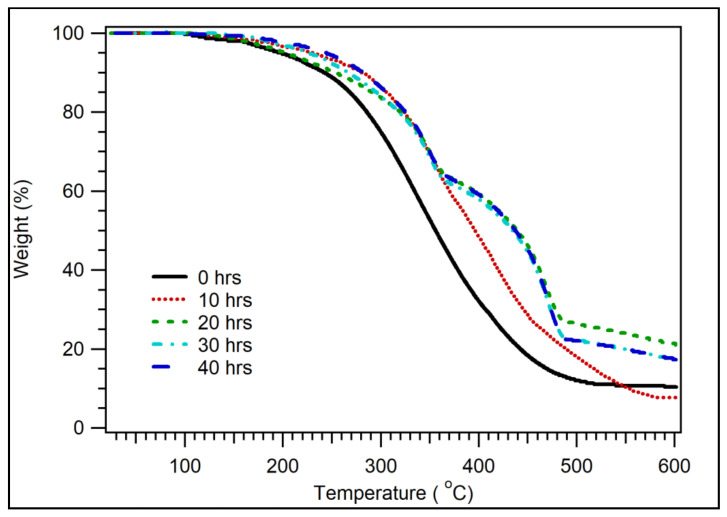
TGA of coconut shell nano-biocomposite for various milling times with 30 wt.% of PP composition.

Figure 8 shows the TGA of coconut shell bio-nanocomposites for 30 wt.% of PP with various durations of milling times. The composite samples started to decompose at a temperature of 150 °C. For 10 and 40 h of milling time, the sample weights were 99.0% and 99.5%, respectively. At a temperature of 200 °C, the sample weight for 0 h milling time remained 94.8%, while it remained 97.8% for 40 h milling time. For sample weight 95%, the decomposition temperature was 199 °C for 0 h milling time. As the milling duration was increased to 10 h, the decomposition temperature increased to 225 °C. It increased to 243 °C for 40 h of milling time. In general, as the duration of milling time was increased, the decomposition of temperature increased, as shown in Figure 8 and Table 6. For 20, 30, and 40 h of milling time, there was a second peak observed at 360 °C. This peak was related to the decomposition of cellulose. At 500 °C, the residual samples were 10–30% of the weight. As shown in Figure 8, as milling time increased (reduced the particle size), the decomposition temperature increased significantly, especially from 0 to 10 h of milling time. The decomposition temperature of biocomposite increased significantly as the milling duration increased because the bond between the filler and adhesive increased with decreasing particle size (increasing milling duration).

**Figure 9:**
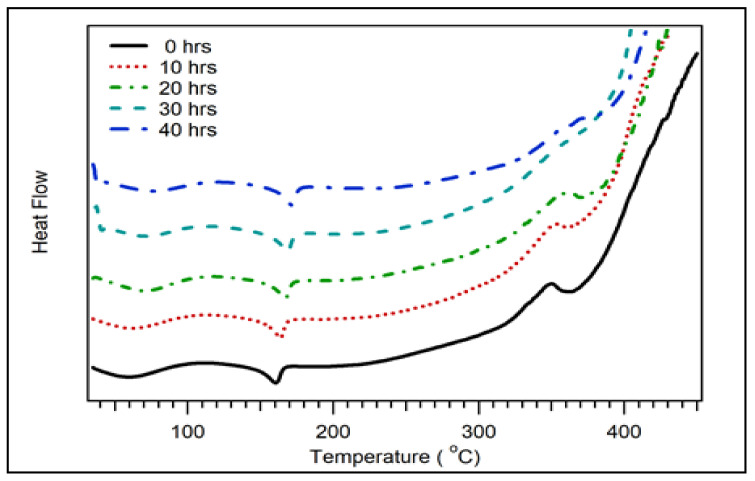
DSC of coconut shell nano-biocomposite for various milling times with 30 wt.% of PP composition.

Figure 9 displays the DSC curves for various milling times with 30 wt.% of PP. The glass transition temperature (Tg) of biocomposite was observed at 60 °C, which increased as the milling times were increased. The melting temperature (Tm) of biocomposite also increased with the milling duration from 0 to 40 h. The depolymerisation temperature (Td) was observed at 350 °C, which increased with the milling times. The summary of the DSC data of coconut shell biocomposite for various milling duration is listed in Table 7. The Tg of biocomposite increased from 60 °C to 75 °C as the milling duration increased from 0 to 40 h. The Tm also increased from 160 °C to 172 °C. The increasing value of glass transition, melting, and depolymerisation temperatures were due to improved bond strength between filler and matrix for nanocomposites. Consequently, the thermal stability of biocomposite improved.

#### 3.2.4. Morphological Properties

The SEM and AFM images of biocomposite for various milling times with 30 wt.% of PP composition are displayed in Table 8. For 0 h duration of milling, some agglomerations and micro-voids were observed. Some coconut shell particles were detached from the matrix (PP) because of weak bonding between the coconut shell and PP. As the milling duration was increased (10–40 h), the number of agglomerations and micro-voids decreased. The surface became smooth and denser, indicating good miscibility between the coconut shell particles with PP. Consequently, the density of biocomposite increased; the thickness swelling and porosity decreased.

**Figure 10:**
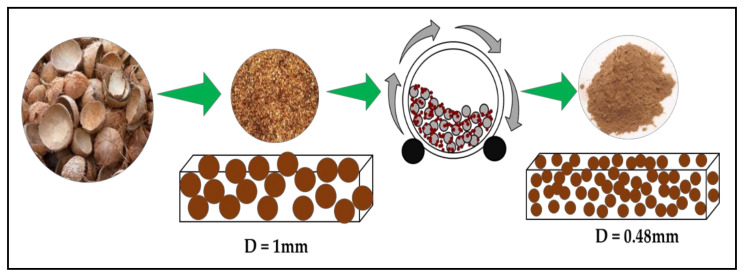
Schematic of reducing the size of filler (particle) from one to nine particles.

Reducing the size of the filler will increase the surface area interaction between the filler and matrix. Assume the diameter of filler (particle) is 1 mm. Then, the volume and surface area of this particle is π/6 mm^3^ and π mm^2^, respectively. Suppose the particle is ground into nineother particles, as shown in Figure 10. Assuming that the total volume of the milled particles is the same as the previous volume, the diameter of the particles after grinding is obtained at 0.48 mm. Then, the total surface area of nineparticles is found to be 2.1π mm^2^. Thus, the surface area interaction between fillers (particles) after grinding becomes twice as before. As a result, the bonding between fillers (particles) and the matrix increases. This simulation can explain why the mechanical and thermal properties of the biocomposite from this study increase with milling time. The bonding between coconut shell particles and PP increased as the milling duration increased. The flexural strength, flexural modulus, and compressive strength of the biocomposite increased. The thermal properties of biocomposite also improved.

This study measured the physical properties, mechanical properties, thermal properties, and morphological properties of coconut shell biocomposite. The results show a close relationship between physical properties and other properties. If the physical properties increase, the mechanical and thermal properties also increase. The morphology of the biocomposite is getting better. All of this has a lot to do with particle size. In other words, particle size becomes important in producing an excellent composite in the future.

## 4. Conclusions

The coconut shell nano-biocomposites using waste polypropylene plastic packaging as a matrix have been successfully prepared and characterised. The physical, mechanical, and thermal properties of biocomposite were dependent on the composition of polypropylene. Instead of increasing the PP composition, the properties of the biocomposite can be improved by reducing the particle size of the coconut shell (increasing the duration of milling). The physical and mechanical properties improved from 0 to 40 h of milling times (density increased from 0.9 to 1.02 g/cm^3^; thickness swelling decreased from 3.4 to 1.8%; porosity decreased from 7.0 to 3.0%; flexural strength increased from 8.19 to 12.26 MPa; flexural modulus increased from 1.67 to 2.87 GPa, and compressive strength increased from 16.00 to 27.20 MPa). Similarly, the thermal properties of biocomposite also improved as the particle size reduced. The degradation temperature increased as the milling duration increased from 0 to 40 h. The glass transition temperature of biocomposite increased slightly. The melting temperature increased significantly from 160 to 170 °C as the milling duration increased from 0 to 40 h. The depolymerisation occurred at 350 °C, which also increased with milling duration. The improvement properties of the biocomposite were due to the increase of bond strength between filler and matrix. This finding indicates that nanoparticles play an important role in biocomposite properties. Two important things can be drawn from this study. First, waste plastic packaging can be utilised to fabricate a high-quality biocomposite. Second, the properties of the biocomposite can be improved by reducing the particle size of filler to nanometres without having to increase the adhesive composition.

## Figures and Tables

**Table 1:** The composition of biocomposite sample for 200 mesh coconut particle size.

Sample No.	Particle Size (Mesh)	CSP (wt.%)	PP (wt.%)
A1	200	70 (210 g)	30 (90 g)
A2	200	60 (180 g)	40 (120 g)
A3	200	50 (150 g)	50 (150 g)
A4	200	40 (120 g)	60 (180 g)
A5	200	0 (0 g)	100 (300 g)

**Table 2:** Decomposition temperature of coconut shell biocomposite for various PP compositions.

Sample Weight (%)	Decomposition Temperature (°C)				
	PP 30 wt.%	PP 40 wt.%	PP 50 wt.%	PP 60 wt.%	PP 100 wt.%
95	199	195	202	249	275
90	242	245	260	300	346
80	286	323	339	351	417
50	356	429	441	442	450
20	443	480	471	500	474

**Table 3:** DSC data of coconut shell biocomposite for various PP compositions.

PP Composition (wt.%)	Tg (°C)	Tm (°C)	Td (°C)
30	60	160	350
40	62	161	350
50	57	161	350
60	57	162	346
100	-	164	340

**Table 4:** SEM and AFM images for the various compositions of PP.

Composition of PP	SEM	AFM
30 wt.%	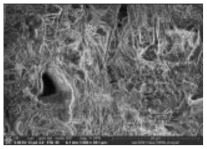	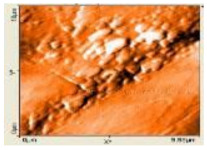
40 wt.%	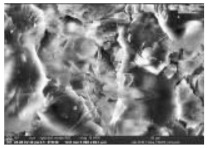	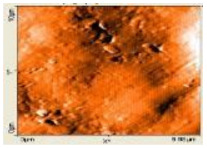
50 wt.%	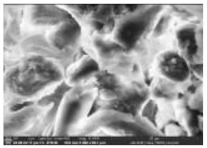	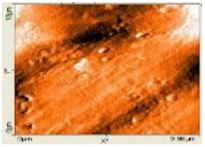
60 wt.%	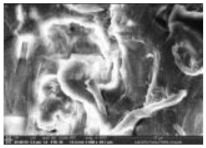	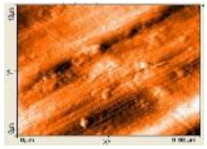
100 wt.%	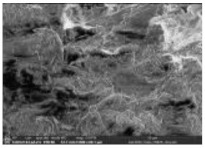	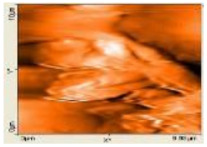

**Table 5:** Crystallite size of coconut shell particles.

Duration of Milling	TEM	XRD Spectra	Crystallite Size (nm)
0 h	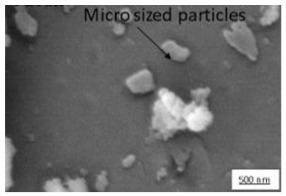	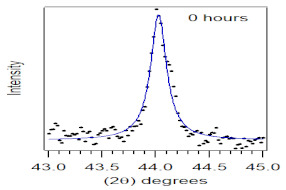	80
10 h	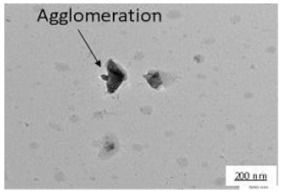	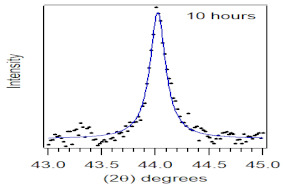	48
20 h	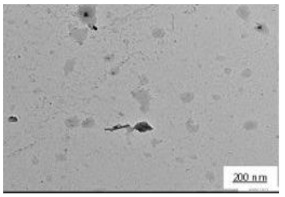	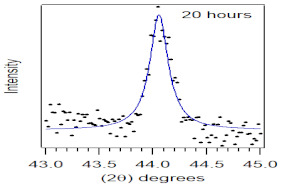	45
30 h	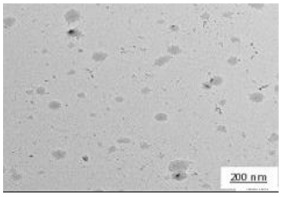	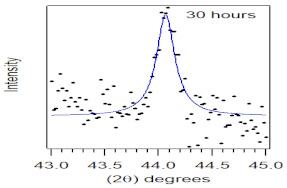	42
40 h	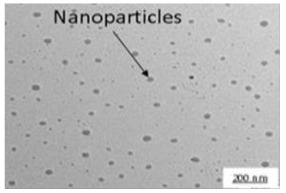	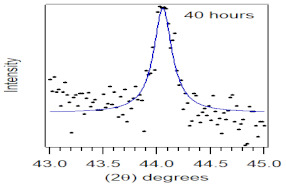	30

**Table 6:** Decomposition temperature of coconut shell biocomposite for various milling times.

Sample Weight (%)	Decomposition Temperature (°C)				
	0 h	10 h	20 h	30 h	40 h
95	199	225	205	226	243
90	242	275	253	267	281
80	286	323	321	318	325
50	356	396	439	435	438
20	443	489	600	544	547

**Table 7:** DSC data of coconut shell biocomposite for various milling times.

Milling Time	Tg (°C)	Tm (°C)	Td (°C)
0 h	60	160	350
10 h	65	162	352
20 h	70	165	359
30 h	70	167	-
40 h	75	172	-

**Table 8:** SEM and AFM images for various milling times, 30 wt.% of PP.

Duration of Milling	SEM	AFM
0 h	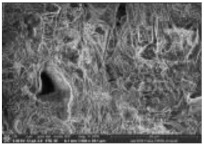	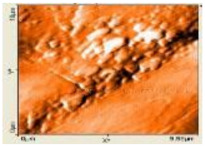
10 h	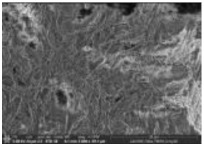	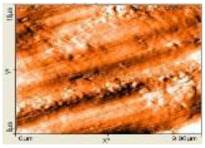
20 h	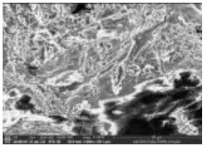	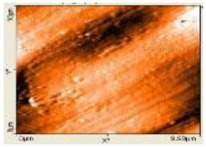
30 h	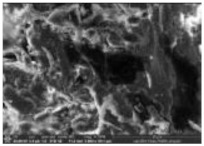	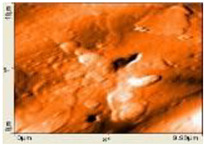
40 h	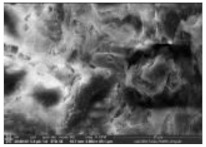	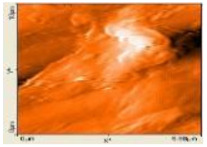

## Data Availability

Not applicable.

## Appendix A

**Table A1:** Analysis of variance for physical properties with various PP compositions.

Physical Properties			Sum of Squares	df	Mean Square	F	Sig.
Density	Levene Statistic df1 df2 Sig.	0.495 4 20 0.740					
	Between Groups		0.044	4	0.011	21.800	0.000
	Within Groups		0.010	20	0.000		
	Total		0.054	24			
Thickness Swelling	Levene Statistic df1 df2 Sig.	0.993 4 20 0.434					
	Between Groups		21.006	4	5.251	54.138	0.000
	Within Groups		1.940	20	0.097		
	Total		22.946	24			
Porosity	Levene Statistic df1 df2 Sig.	0.899 4 20 0.483					
	Between Groups		117.322	4	29.331	155.353	0.000
	Within Groups		3.776	20	0.189		
	Total		121.098	24			

**Table A2:** Analysis of variance for mechanical properties with various PP compositions.

Mechanical Properties			Sum of Squares	df	Mean Square	F	Sig.
Flexural Strength	Levene Statistic df1 df2 Sig.	0.799 4 20 0.540					
	Between Groups		59.230	4	14.808	27.407	0.000
	Within Groups		10.806	20	0.540		
	Total		70.036	24			
Flexural Modulus	Levene Statistic df1 df2 Sig.	1.418 4 20 0.264					
	Between Groups		2.975	4	0.744	36.774	0.000
	Within Groups		0.405	20	0.020		
	Total		3.380	24			
Compressive Strength	Levene Statistic df1 df2 Sig.	0.233 4 20 0.917					
	Between Groups		53.382	4	13.346	56.935	0.000
	Within Groups		4.688	20	0.234		
	Total		58.070	24			

**Table A3:** Analysis of variance for physical properties with various milling times.

Physical Properties			Sum of Squares	df	Mean Square	F	Sig.
Density	Levene Statistic df1 df2 Sig.	1.793 4 20 0.170					
	Between Groups		0.054	4	0.014	21.938	0.000
	Within Groups		0.012	20	0.001		
	Total		0.066	24			
Thickness Swelling	Levene Statistic df1 df2 Sig.	1.388 4 20 0.274					
	Between Groups		8.046	4	2.011	28.571	0.000
	Within Groups		1.408	20	0.070		
	Total		9.454	24			
Porosity	Levene Statistic df1 df2 Sig.	1.152 4 20 0.361					
	Between Groups		72.290	4	18.072	117.353	0.000
	Within Groups		3.080	20	0.154		
	Total		75.370	24			

**Table A4:** Analysis of variance for mechanical properties with various milling times.

Mechanical Properties			Sum of Squares	df	Mean Square	F	Sig.
Flexural Strength	Levene Statistic df1 df2 Sig.	1.298 4 20 0.305					
	Between Groups		48.019	4	12.005	39.028	0.000
	Within Groups		6.152	20	0.308		
	Total		54.171	24			
Flexural Modulus	Levene Statistic df1 df2 Sig.	0.621 4 20 0.653					
	Between Groups		5.723	4	1.431	102.017	0.000
	Within Groups		0.280	20	0.014		
	Total		6.004	24			
Compressive Strength	Levene Statistic df1 df2 Sig.	0.530 4 20 0.715					
	Between Groups		456.262	4	114.065	228.771	0.000
	Within Groups		9.972	20	0.499		
	Total		466.234	24			
